# AT-101 enhances gefitinib sensitivity in non-small cell lung cancer with EGFR T790M mutations

**DOI:** 10.1186/s12885-016-2519-3

**Published:** 2016-07-18

**Authors:** Ren Zhao, Shun Zhou, Bing Xia, Cui-ying Zhang, Ping Hai, Hong Zhe, Yan-yang Wang

**Affiliations:** Department of radiation oncology, General Hospital of Ningxia Medical University, Yinchuan, 750004 Ningxia China; Cancer Institute, Ningxia Medical University, Yinchuan, 750004 Ningxia China; Graduated School, Ningxia Medical University, Yinchuan, 750004 Ningxia China; Department of Radiation Oncology, Hangzhou Cancer Hospital, Nanjing Medical University, Hangzhou, 310000 Zhejiang China

**Keywords:** Non-small cell lung cancer, EGFR TKIs resistance, EGFR T790M mutation, Gefitinib, AT-101, Bcl-2

## Abstract

**Background:**

Although epidermal growth factor receptor tyrosine kinase inhibitors (EGFR TKIs) have become the standard care of patients with advanced EGFR-mutant non-small cell lung cancer (NSCLC), development of acquired resistance is inevitable. A secondary mutation of threonine 790 (T790M) is associated with approximately half of the cases of acquired resistance. Strategies or agents to overcome this type of resistance are still limited. In this study, enhanced antitumor effect of AT-101, a-pan-Bcl-2 inhibitor, on gefitinib was explored in NSCLC with T790M mutation.

**Methods:**

The effect of cotreatment with AT-101 and gefitinib on the viability of NSCLC cell lines harboring acquired T790M mutation was investigated using the MTT assay. The cellular apoptosis of NSCLC cells after cotreatment with AT-101 and gefitinib was assessed by FITC-annexin V/PI assay and Western blots analysis. The potential underlying mechanisms of the enhanced therapeutic effect for AT-101 was also studied using Western blots analysis. The in vivo anti-cancer efficacy of the combination with AT-101 and gefitinib was examined in a mouse xenograft model.

**Results:**

In this study, we found that treatment with AT-101 in combination with gefitinib significantly inhibited cell proliferation, as well as promoted apoptosis of EGFR TKIs resistant lung cancer cells. The apoptotic effects of the use of AT-101 was related to the blocking of antiapoptotic protein: Bcl-2, Bcl-xl, and Mcl-1 and downregrulation of the molecules in EGFR pathway. The observed enhancements of tumor growth suppression in xenografts supported the reverse effect of AT-101 in NSCLC with T790M mutation, which has been found in in vitro studies before.

**Conclusions:**

AT-101 enhances gefitinib sensitivity in NSCLC with EGFR T790M mutations. The addition of AT-101 to gefitinib is a promising strategy to overcome EGFR TKIs resistance in NSCLC with EGFR T790M mutations.

## Background

Lung cancer is a major health problem with a generally grim prognosis [1]. Non-small cell lung cancer (NSCLC), the most common lung cancer, is often diagnosed as advanced stage and the opportunity for surgical resection is lost. For these advanced stage NSCLC patients, whose tumors harbor epidermal growth factor receptor (EGFR) activating mutation, EGFR tyrosine kinase inhibitors (TKIs) such as gefitinib and erlotinib exert potential therapeutic effects [2–4]. However, despite an initial dramatic response to TKIs, acquired resistance develops in most NSCLC patients with EGFR mutations after 10–14 months of treatment [5–7]. Multiple mechanisms of acquired resistance to TKIs have been identified, including secondary EGFR mutation in threonine 790 (T790M), MET amplification, human epidermal growth factor receptor2 (HER2) amplification, conversion from NSCLC into small cell lung cancer, and loss of phosphatase and tensin homolog (PTEN) [8]. More and more evidences suggests that at least half of the EGFR TKIs resistance is caused by acquired EGFR T790M mutation [9, 10]. To date, there is no effective management methods for patients with acquired T790M mutation. Therefore, strategies or agents to overcome EGFR TKIs resistance is critical to prolong the survival of patients with NSCLC [11].

**Fig. 1:**
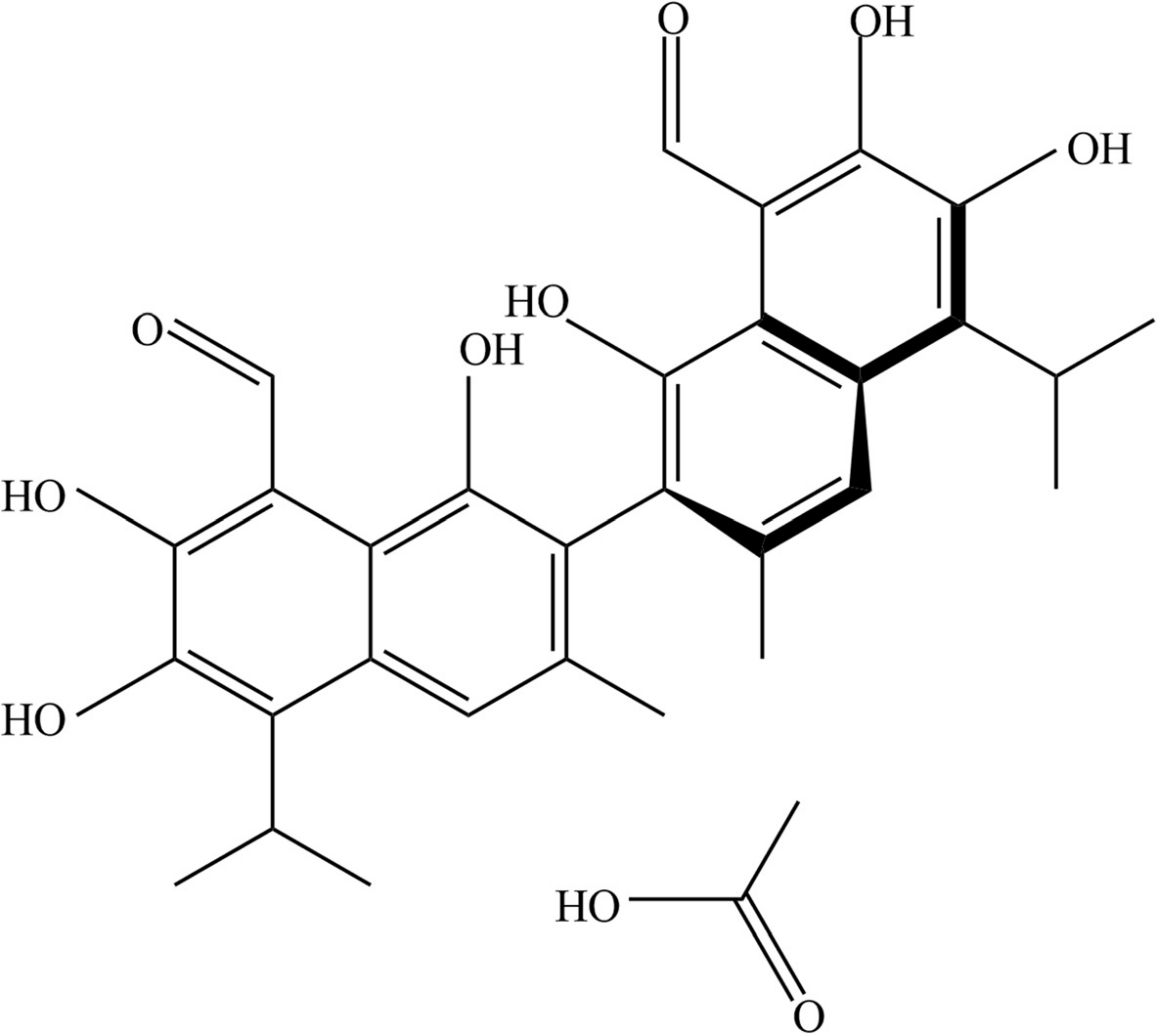
Chemical structures of R-(−)-gossypol (AT-101)

Zou et al. revealed that the knockdown B-cell lymphoma 2 (Bcl-2) gene expression by siRNA could reverse acquired T790M mutation in EGFR TKIs resistant H1975 cell line [12]. AT-101 (ie, R-(−)-gossypol acetic acid, Fig. 1), a pan-Bcl-2 inhibitor, has shown antitumor activity in several cancer cell lines [13–15]. Thus, it is reasonable to think that AT-101 would enhance the activity of EGFR TKIs leading to apoptosis and growth inhibition of resistant cells in which EGFR TKIs have failed to completely abolish EGFR activity. We investigated whether the combination of AT-101 and gefitinib could reverse EGFR TKIs resistance associated with T790M mutation in NSCLC cells.

## Methods

### Cell lines and reagents

PC-9 gefitinib-resistant cells (PC-9-GR) was kindly provided by Dr. Bing Xia at Hangzhou Cancer Hospital (Hangzhou, China) [16]. H1975 cell lines was obtained from the American Type Culture Collection (Manassas, VA, USA). Cells were cultured in standard RPMI-1640 media (Invitrogen, Carlsbad, CA, USA) supplemented with 10 % heat-inactivated fetal bovine serum (Invitrogen, Carlsbad, CA, USA), 2 mM l-glutamine, and 1 % penicillin/streptomycin at 37 °C in a humidified incubator with 5 % CO_2_. AT-101, ABT-263, and gefitinib were purchased from Sigma (St. Louis, MO, USA). Both reagents were dissolved in dimethyl sulfoxide (DMSO), stored at−80 °C, and diluted in culture medium for described experiments.

### Cell viability assay

Cytotoxicity of AT-101 and/or gefitinib was evaluated by 3-(4, 5-dimethylthiazol-2-yl)-2, 5-diphenyl tetrazolium bromide (MTT) (Sigma Chemical Co., St. Louis, MO, USA). Briefly, PC-9-GR and H1975 cells were plated in 96-well plates and cultured overnight for attachment. The next day, the cells were treated with various concentrations of AT-101 and/or gefitinib for 24 h. Following drug treatment, 0.5 mg/mL of MTT was added to the media. The reaction was terminated by the addition of 100 μl DMSO. The optical density of the MTT formazan product was read at 490 nm on a microplate reader. Absorbance values were expressed as a percentage of that for untreated cells. The combination effect of gefitinib and ABT-263, a more specific Bcl-2 inhibitor, was also evaluated by MTT assay.

### Cellular apoptosis analysis

The PC-9-GR and H1975 cell lines undergoing early/late apoptosis were analyzed by annexinV-FITC and PI staining. Experiments were performed by using an annexin V-FITC apoptosis detection kit (BD Biosciences, USA) according to the manufacturer’s protocol. In brief, cells were harvested and washed, and then incubated with Annexin V-FITC and propidiumiodide (PI) in 1 × binding buffer for 15 min at room temperature in the dark. The populations of apoptotic cells were determined using a Becton Dickinson FACScan cytofluorometer. Both early apoptotic (Annexin V-positive and PI-negative) and late apoptotic (Annexin V-positive and PI-positive) cells were included as total apoptosis.

### Western blots analysis

Total proteins from PC-9-GR and H1975 cells were extracted with RIPA buffer containing a protease inhibitor cocktail. Protein samples were quantified using a Bio-Rad DC protein assay kit II (Bio-Rad, Hercules, CA, USA). Thirty microgram protein of each sample was subjected to SDS-PAGE (7–12 % SDS-acrylamide gel) and the separated proteins were transferred to polyvinylidene difluoride (PVDF) membrane (Millipore, Bedford, MA, USA) for 2 h at 100 mA. After blocking in 5 % BSA-TBST or milk-TBST, membranes were blotted with total EGFR (Cell Signaling Technology, Beverly, MA, USA), phospho-EGFR (Tyr1068, Cell Signaling Technology, Beverly, MA, USA), total Akt (Cell Signaling Technology, Beverly, MA, USA), phospho-Akt (Ser473; Cell Signaling Technology, Beverly, MA, USA), total Erk (Cell Signaling Technology, Beverly, MA, USA), phospho-Erk (Thr202/Tyr204; Cell Signaling Technology, Beverly, MA, USA), Bcl-2 (Santa Cruz, CA, USA), B-cell lymphoma-extra large (Bcl-xl) (Santa Cruz, CA, USA), Myeloid cell leukemia 1 (Mcl-1) (Santa Cruz, CA, USA), cleaved caspase-3 (Santa Cruz, CA, USA) and β-actin (Santa Cruz, CA, USA) followed by horseradish peroxidase-conjugated goat antibodies to rabbit or mouse (Santa Cruz, CA, USA) immunoglobulin G. Expression was visualised by using enhanced chemiluminescence (ECL) (GE Health Care Bio-Sciences, NJ, USA). Protein level was normalized to the matching densitometric value of the internal control.

### Xenograft studies

Laboratory animal handling and experimental procedures were performed in accordance with the requirements of Provisions and General Recommendation of Chinese Experimental Animals Administration Legislation. The research project was examined and certified by the Ethics Committee of the General Hospital of Ningxia Medical University. PC-9-GR cells (1 × 10^6^ cells · 0.1 ml per mouse) were inoculated subcutaneously into the right front axilla of female athymic BALB-c/nu mice at 5 to 6 weeks of age (Charles River, Beijing, China). Treatment of 6 mice per group was started when the tumors had reached a volume of 150 to 200 mm^3^ with vehicle control, gefitinib (50 mg/kg, 5 days a week), AT-101 (35 mg/kg, 5 days a week), or gefitinib plus AT-101. Both drugs were orally administered. Tumor volume was determined from caliper measurements of tumor length (L) and width (W) according to the formula LW^2^/2. Both tumor size and body weight were measured twice per week. Immunohistochemical analysis was performed on formalin-fixed, paraffin-embedded tissue sections staining for Ki-67 (Cell Signaling Technology, Beverly, MA, USA) and cleaved caspase-3 (Santa Cruz, CA, USA).

### Immunohistochemical (IHC) staining

Sacrificed tumors were fixed, embedded in paraffin, and sectioned (4 μm). These tissue sections were then dried, deparaffinized and rehydrated, and after quenching endogenous peroxidase activity and blocking non-specific binding sites. After that, sections were incubated overnight at 4 °C with 1:100 dilution of primary antibody directed against Ki-67 and cleaved caspase-3 and followed by a 30 min incubation with a secondary antibody. The immunolabeled sections were visualized with 3,3’-diaminobenzidine and counterstained with hematoxylin. Quantitative analysis of section staining was done by counting immunopositive cells in 5 arbitrarily selected fields at × 400 magnification.

### Statistical analysis

All statistical calculations were performed by Statistical Package for the Social Sciences (SPSS) 13.0 software (Chicago, IL, USA). Results were representative of three independent experiments unless stated otherwise. In vitro results are expressed as mean ± SD and in vivo results are expressed as mean ± SE. One-way Analysis of Variance (ANOVA) test was used to analyze significance between groups. A *P* value of <0.05 was considered statistical significant. The synergistic effect of AT-101 and gefitinib was assessed by the Biosoft CalcuSyn program (Ferguson, MO, USA). The combination index (CI) was used to express synergism (CI < 1), additive effect (CI = 1), or antagonism (CI > 1).

## Results

### AT-101 enhances gefitinib sensitivity in NSCLC cells with EGFR T790M mutations

**Fig. 2:**
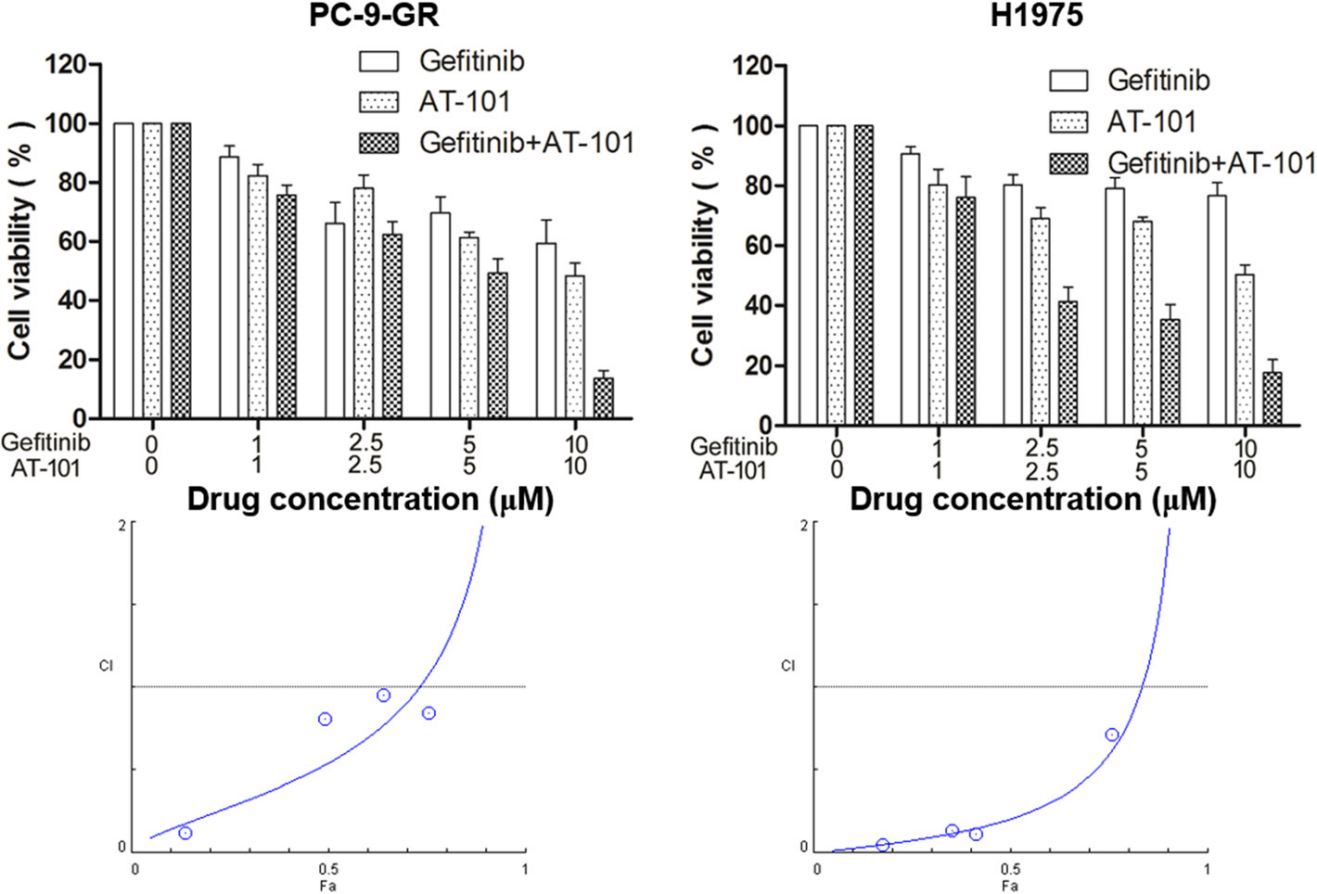
Effect of combination treatment with AT-101 and gefitinib on cell viability of NSCLC cells with the T790M mutation. Top, cell viability was determined by the MTT assay. Data are shown as mean ± SD of three independent experiments. Bottom, the combined effect of the two drugs was evaluated on the basis of the combination index (CI) of each drug fraction

**Fig. 3:**
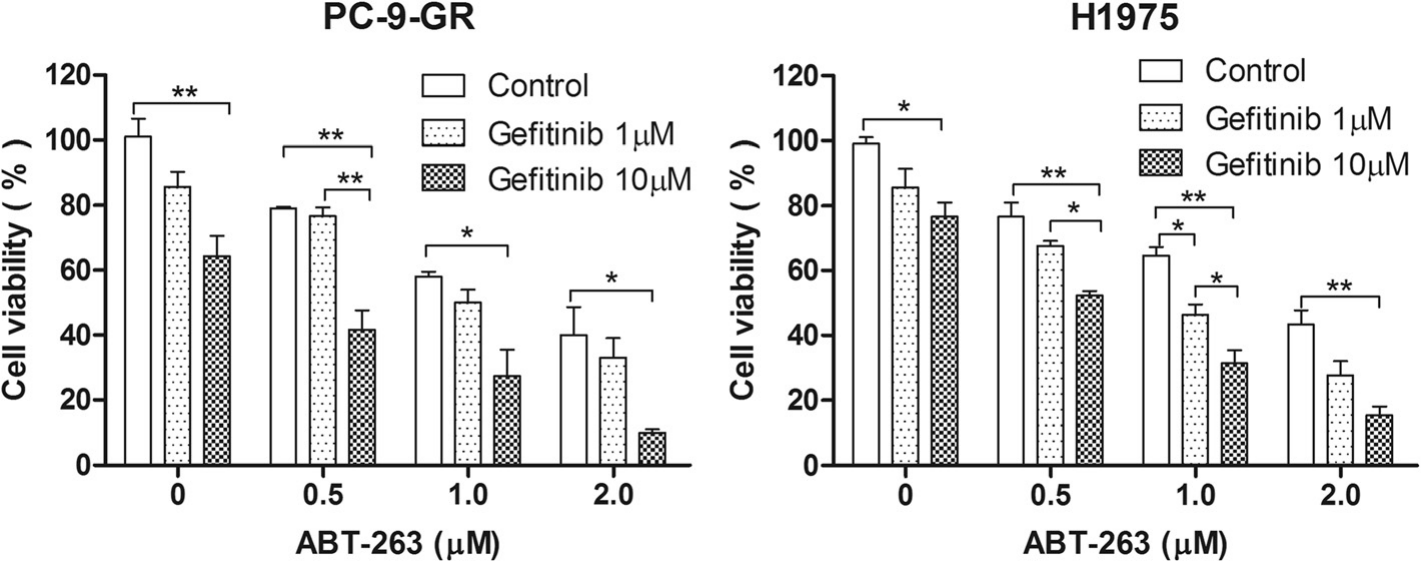
Effect of combination treatment with ABT-263 and gefitinib on cell viability of NSCLC cells with the T790M mutation. PC-9-GR and H1975 cells were treated with different concentrations of gefitinib in the absence or presence of ABT-263 for 24h, and viability was then measured using the MTT assay. The data represent the mean ± SD of three independent experiments. *, *P* < 0.05; **, *P* < 0.01; ***, *P* < 0.001

The inhibition of proliferation of combination treatment with AT-101 and gefitinib in human NSCLC cells with an EGFR T790M mutation was measured by MTT assay. PC-9-GR and H1975 cells were exposed to individual agents or a combination of AT-101 with gefitinib. AT-101 or gefitinib alone could reduce the viability of PC-9-GR and H1975 cells in a small amount with given concentration. However, cotreatment with AT-101 could enhance the ability of EGFR-TKIs to induce growth inhibition. Furthermore, the combined effect of the two drugs was also evaluated on the basis of the CI. The combination of AT-101 and gefitinib manifested a synergistic inhibitory effect (CI of <1.0) on the growth of both PC-9-GR and H1975 cells (Fig 2). In order to further assess the Bcl-2 inhibition effect on EGFR TKIs resistance, the combination effect of gefitinib and ABT-263, a more specific Bcl-2 inhibitor, was also evaluated. The results showed that ABT-263 also enhanced the anti-proliferation effect of EGFR inhibitor gefitinib in both PC-9-GR and H1975 cells (Fig. 3).

**Fig. 4:**
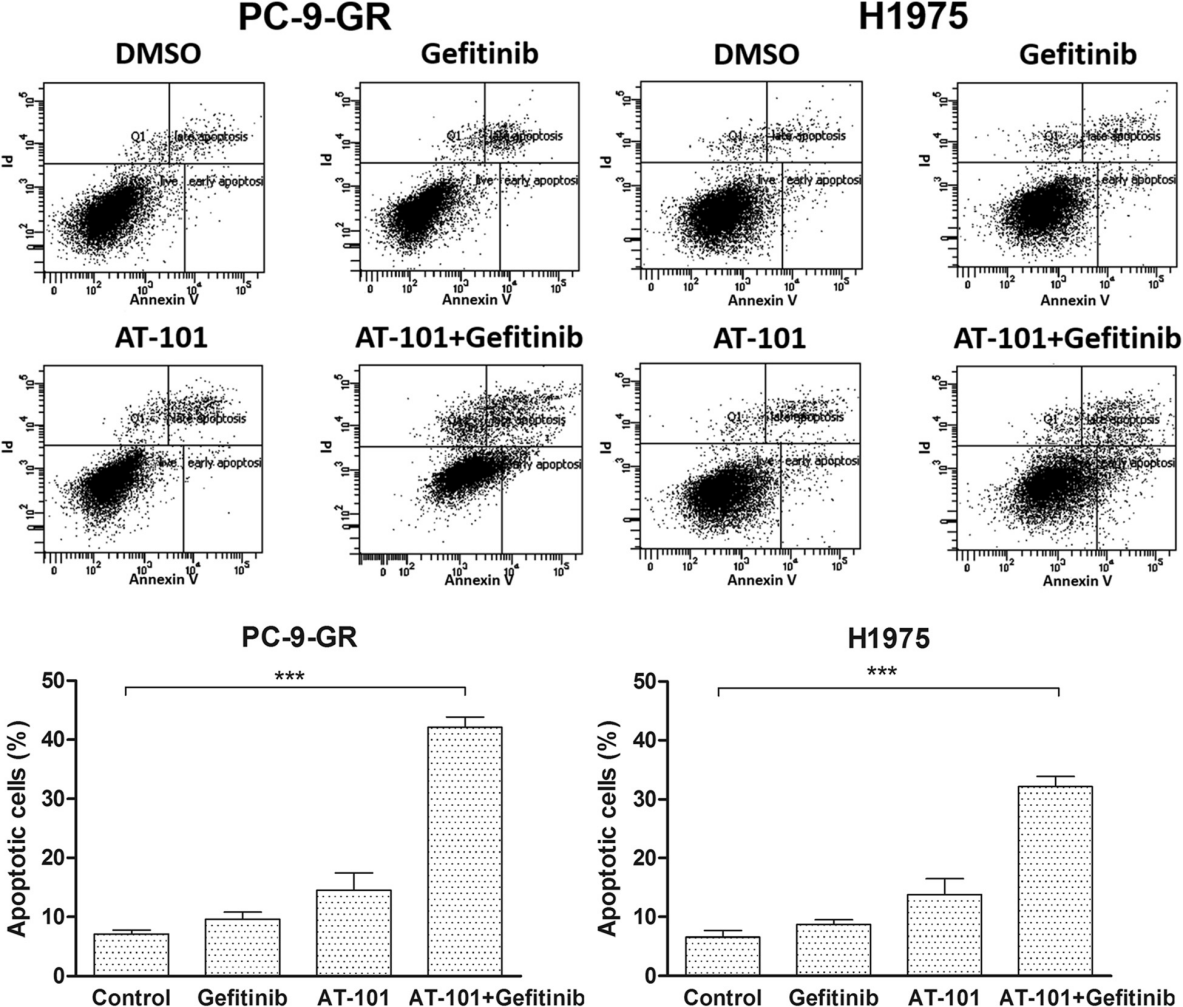
Effect of combination treatment with AT-101 and gefitinib on apoptosis of NSCLC cells with the T790M mutation. Cells were incubated with 5 μM AT-101 and/or 1 μM gefitinib for 24h, and apoptosis was assessed by Annexin V/PI staining and fluorescence activated cell-sorting (FACS) analysis. Columns representing the flow cytometry data are presented on the top. Bars represent the mean ± SD of three independent experiments. ***, *P* < 0.001

To explore whether the observed growth inhibition was due to enhanced apoptosis, the proportion of apoptotic cells was determined using annexin V-PI staining. Apoptotic cells were markedly increased in both PC-9-GR and H1975 cells with the combination treatment of AT-101 with gefitinib when compared with either AT-101 or gefitinib treatment alone (Fig. 4).

### The underlying mechanism of the enhance effect of AT-101 on gefitinib in NSCLC cells with EGFR T790M mutations

To elucidate the mechanism of apoptosis induced by AT-101 and gefitinib, cell lysates were evaluated by immunoblotting in PC-9-GR and H1975 NSCLC cells.

As AT-101 is a pan-Bcl-2 inhibitor, the expression level of Bcl-2, Bcl-xl and the apoptosis related protein cleaved caspase-3 were initial tested. Our results showed that the combination of AT-101 and gefitinib suppressed the expression of Bcl-2 and Bcl-xl. Moreover, combination of AT-101 and gefitinib led to a marked increase in the expression of cleaved caspase-3. These results indicate that AT-101 and gefitinib play a major role in enhancing caspase-dependent apoptosis through inhibition of antiapoptotic proteins in NSCLC cells with the T790M mutation.

To further understand the mechanism by which AT-101 restored the antitumor activities of the EGFR TKIs, the activities of EGFR and its downstream molecules were examined. As expected, the inhibitory effect of single treatment with AT-101, gefitinib, on EGFR, Akt and Erk activities was modest, whereas the combination of AT-101 and gefitinib substantially suppressed EGFR, Akt and Erk activities.

**Fig. 5:**
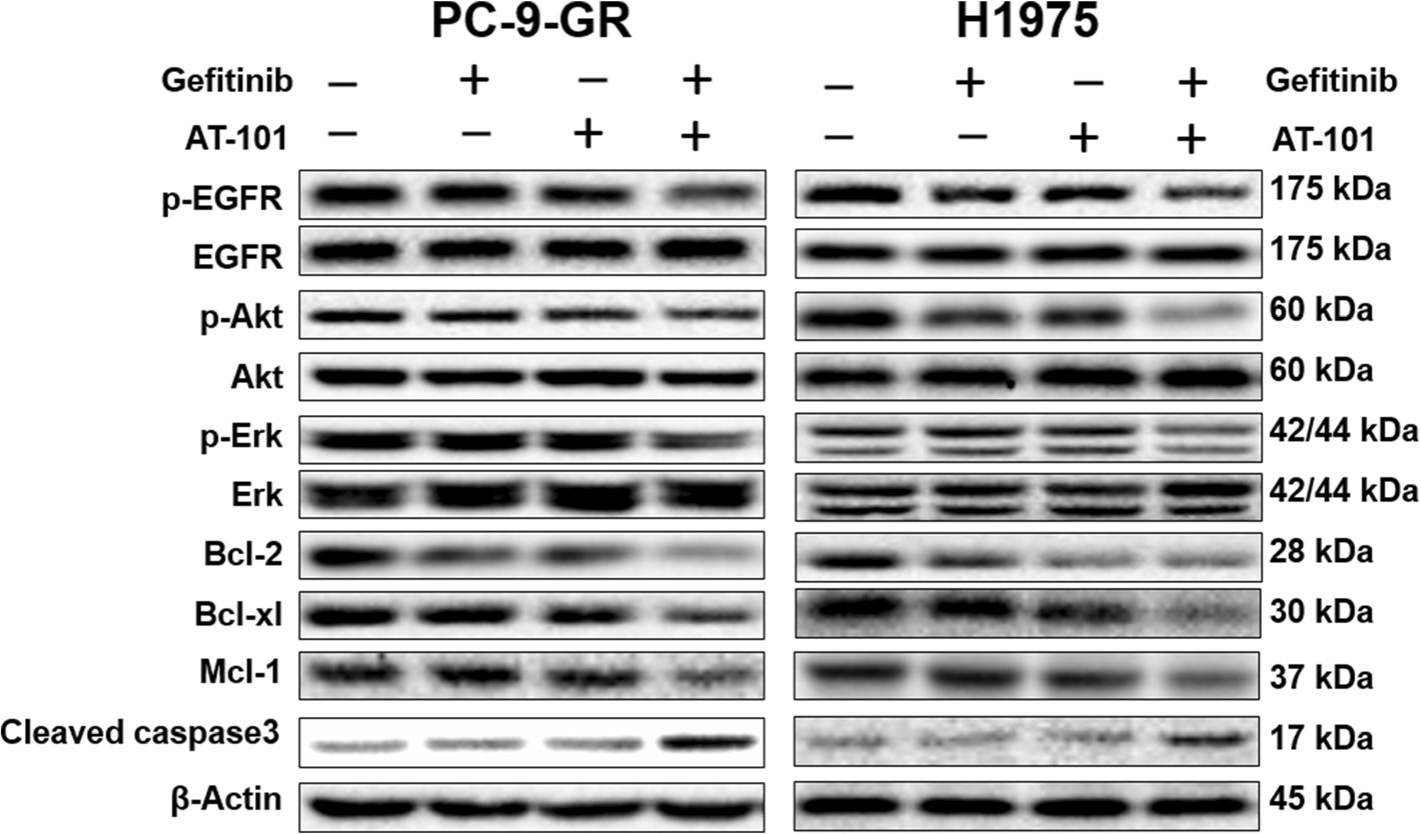
The enhanced therapeutic effect of AT-101 on gefitinib in NSCLC cells with the T790M mutation through inhibition of antiapoptotic proteins and molecules of EGFR pathway. PC-9-GR and H1975 cells were treated with 5 μM AT-101, 1μM gefitinib or a combination as indicated for 12 hours. Western blot analysis was performed to detect the expression of p-EGFR, EGFR, p-Akt, Akt, p-Erk, Erk, Bcl-2, Bcl-xl, Mcl-1, and cleaved caspase 3. β-actin was used as the loading control

Taken together, these results suggest that AT-101 treatment may have advantages in NSCLC cells with T790M on the basis of the ability to inhibit the activities of antiapoptotic proteins, and also EGFR and downstream signaling molecules (Fig. 5)

.

### Addition of AT-101 to gefitinib enhances antitumor activity of EGFR TKIs resistant tumor xenografts in vivo

**Fig. 6:**
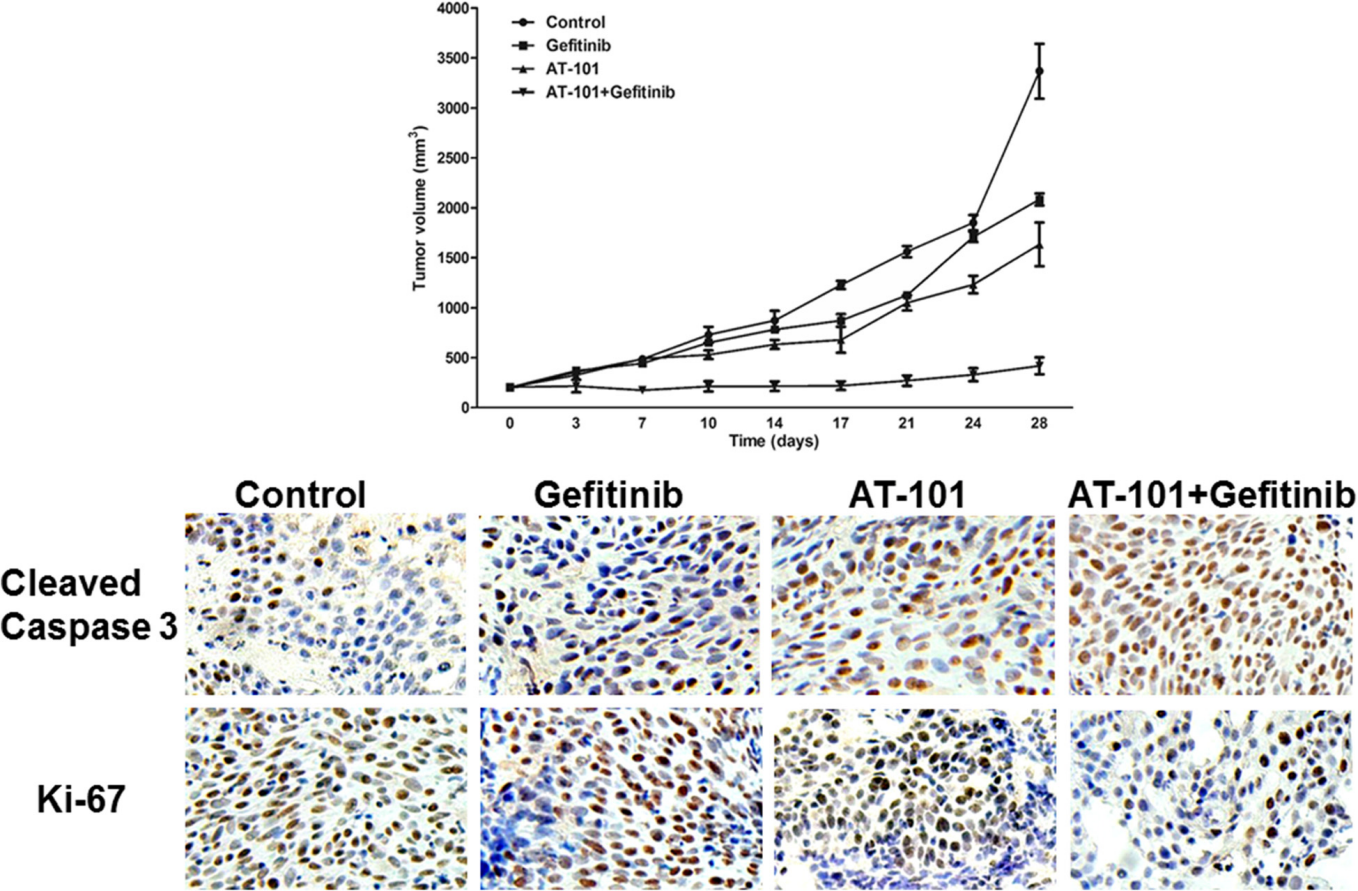
Addition of AT-101 to gefitinib enhances antitumor activity of EGFR TKIs resistant tumor xenografts in vivo. PC-9-GR cells (1 × 106 cells·0.1 ml per mouse) were inoculated subcutaneously into the right front axilla of nude mice. Mice bearing tumors of 150 to 200 mm3 in size were then randomly divided into groups that received either vehicle control, gefitinib (50 mg/kg, 5 days a week), AT-101 (35 mg/kg, 5 days a week), or gefitinib plus AT-101. The tumor volumes were measured twice per week. Each treatment group consisted of six mice. The data shown represent mean ± SD. The tumors were resected from the sacrificed nude mice and fixed in 10% neutral buffered formalin. Immunohistochemistry assays were conducted for cleaved caspase 3 and Ki-67

To further evaluate the antitumor efficacy of the combination with AT-101 and gefitinib, athymic BALB-c/nu mice bearing PC-9-GR implanted xenografts were treated by oral gavage with AT-101 and gefitinib, both alone and in combination, for the duration of the experiment. As shown in Fig. 6, treatment of the PC-9-GR NSCLC xenograft model with AT-101 or gefitinib alone resulted in modest inhibitions of tumor growth compared with the vehicle-treated control. However, the combination of AT-101 with gefitinib resulted in significant tumor regression. The enhanced antitumor activity of the combined treatment of AT-101 and gefitinib was also confirmed by immunohistochemical (IHC) staining for Ki-67, a marker for cell proliferation. Consistent with in vitro observations, staining for cleaved caspase-3 was markedly increased upon combined administration of AT-101 and gefitinib. These results support the suggestion that the addition of AT-101 to EGFR TKIs may reverse T790M-mediated resistance through the restoration of the ability of EGFR TKIs to downregulate EGFR signals, which induces cell growth inhibition.

## Discussion

Although NSCLC with EGFR mutation display significant responses to EGFR TKIs, cancer cells eventually become resistant to the treatment and median duration of response is about 10 to 14 months [5–7]. Approximately 50 % of cases with acquired resistance to EGFR TKIs therapy are related to T790M mutation [9, 10]. The T790M substitution is known to disturb the proper binding of the drug to the ATP pocket of EGFR and to restore the affinity for ATP at the level of wild type EGFR [17, 18]. In order to solve this problem, new strategies have been developed against resistant mutations of EGFR, such as irreversible binding to the ATP pocket of EGFR and the selective targeting of T790M-harboring receptors [11, 19, 20]. Although these irreversible inhibitors are more potent than gefitinib against T790M [21], more studies are warranted to further demonstrate the efficacy and safety of these new generation of EGFR TKIs in the phase III clinical trials.

Bcl-2 family proteins are known to be key regulators of apoptotic cell death [22, 23]. Overexpression of antiapoptotic Bcl-2 family members such as Bcl-2, Bcl-xl, and induced myeloid leukemia cell differentiation protein Mcl-1 (Mcl-1) has been identified in a number of EGFR TKIs resistance NSCLC cell lines and is considered as a therapeutic target for reversing the resistance. Zou et al. demonstrated downregulation of Bcl-2 by siRNA could reverse drug resistance to gefitinib in H1975 lung cancer cell line harboring T790M mutation. AT-101, known as R-(−)-gossypol, has been shown to possess widespread antiproliferative activity against several tumour cells through inhibition of the Bcl-2 family members [12]. Based on these findings, we hypothesize that the combination of AT-101 and gefitinib could overcome EGFR TKIs resistance associated with T790M mutation in NSCLC.

The results of this study clearly show that AT-101 enhances sensitivity to EGFR TKIs gefitinib in both PC-9-GR and H1975 cells. Treatment with AT-101 in combination with gefitinib significantly inhibited cell proliferation, as well as promoted apoptosis of EGFR TKIs resistant lung cancer cells. We found that the enhanced effect of AT-101 was associated with the suppression of the antiapoptotic protein: Bcl-2 and Bcl-xl. To further explore the mechanisms involved in the combination therapy, we investigated the inhibitory effect of combination therapy on the EGFR signaling pathway. There are two major downstream signalling pathways of EGFR, which includes the Ras/Raf/MAPK/Erk pathway for cell proliferation and PI3K/Akt/mTOR pathway for cell survival [24, 25]. We found that AT-101-induced degradation of mutant EGFR influenced both Ras/Raf/MAPK/Erk and PI3K/Akt/mTOR pathways. The downregulation of EGFR pathway may be another mechanism of the activation of apoptosis. Moreover, a marked increase in cleaved caspase-3 staining accompanied by a reduction in Ki-67 staining was observed in the xenograft lung cancer samples treated with AT-101 plus gefitinib. The observed enhancements of tumor growth suppression in xenografts further supported the reverse effect of AT-101 in gefitinib-resistant PC-9-GR cells. These data suggest that combined treatment with an inhibitor of Bcl-2 and gefitinib is a potential therapeutic strategy for treatment of patients with acquired resistance to first generation EGFR TKIs due to secondary EGFR T790M mutation.

## Conclusions

We revealed that the combined treatment with AT-101 and gefitinib induced additional cell death in vitro and in vivo, overcame the acquired resistance to EGFR TKIs in T790M mutant lung cancer, and that cell death is mediated through inhibition of bcl-2, downregulation of phosphorylation of EGFR, Akt, and Erk and induction of cell apoptosis. These findings provide a new concept for the development of novel therapeutic approaches in the treatment of refractory and relapsed patients who are no longer sensitive to EGFR TKIs. However, the activity of this cotreatment strategy in the clinical setting remains to be clarified.
